# Effects of Modified Dietary Fiber from Fresh Corn Bracts on Obesity and Intestinal Microbiota in High-Fat-Diet Mice

**DOI:** 10.3390/molecules28134949

**Published:** 2023-06-23

**Authors:** Ningning Geng, Ying Li, Yan Zhang, Hongjuan Wang, Jiangfeng Song, Lijun Yu, Caie Wu

**Affiliations:** 1 Institute of Agro-Product Processing, Jiangsu Academy of Agricultural Sciences, Nanjing 210014, China; 2 College of Light Industry and Food Engineering, Nanjing Forestry University, Nanjing 210037, China; 3 College of Food Science and Technology, Nanjing Agricultural University, Nanjing 210095, China

**Keywords:** fresh corn bracts, modified dietary fiber, obese mice, intestinal microbiota

## Abstract

The effects of insoluble dietary fiber from fresh corn bracts modified by dynamic high-pressure micro-fluidization (DHPM) on the pathological characteristics of obesity, intestinal microflora distribution and production of short-chain fatty acids in high-fat-diet C57BL/6 mice were evaluated. The results show that the DHPM-modified dietary fiber from fresh corn bracts significantly reduces weight gain, insulin resistance and oxidative damage caused by a high-fat diet, and promotes the production of SCFAs, especially acetic acid, propionic acid and butyric acid. These modified dietary fibers also change the proportion of different types of bacteria in the intestinal microflora of mice, reduce the ratio of *Firmicutes* and *Bacteroidota* and promote the proliferation of *Bifidobacteriales*. Therefore, the DHPM-modified dietary fiber from fresh corn bracts can be used as a good intestinal microbiota regulator to promote intestinal health, thereby achieving the role of preventing and treating obesity.

## 1. Introduction

Dietary fiber is a carbohydrate polymer composed of multiple monomer units that are not hydrolyzed by endogenous enzymes in the human small intestine, mainly including soluble dietary fiber and insoluble dietary fiber [1]. The digestion and decomposition of dietary fiber are mainly carried out in the large intestine. Intestinal microorganisms in the large intestine, including bacteria, archaea and fungi, selectively decompose, ferment and utilize dietary fiber [2]. These intestinal microorganisms can secrete different enzymes in the process of reproduction and metabolism to hydrolyze dietary fiber, especially soluble dietary fiber, and thus become nutrients for the growth and proliferation of intestinal bacteria. These products used by the intestinal bacteria, in turn, affect the type and quantity of intestinal microbiota [3]. Changes in the intestinal microbiota may affect body weight, blood glucose, blood lipids and insulin sensitivity [4]. Zhou et al. [5] studied the effect of microbiotic changes on insulin resistance and dyslipidemia in mice. The experiment used whole-wheat oatmeal to feed five-week-old male C57BL/6J mice for 8 weeks. It was found that Prevotellaceae, Lactobacillaceae and Alcaliginaceae were increased in the intestinal microbiota, and the insulin sensitivity and glucose level were improved. Wang et al. [6] originated that soluble dietary fiber reduced weight gain and the excessive accumulation of white adipose tissue in obese mice that were fed a high-fat diet. According to 16S rRNA sequencing, soluble dietary fiber restored the diversity of gut microbiota. However, most natural dietary fiber is insoluble, and its functional activity and physico-chemical properties are not as good as those of soluble dietary fiber [2]. Therefore, it is very important to modify insoluble dietary fiber and clarify its functional activity.

Fresh corn, mainly referring to fruit corn such as sweet corn and waxy corn, is harvested in the late stage of milk ripening and early stage of wax ripening. It is favored by consumers because of its sweet and delicious taste, unique flavor and rich nutrition. It contains carbohydrates, proteins, fats, carotenoids, as well as nutrients such as riboflavin and vitamins, which have great benefits in preventing heart disease and cancer [7]. Fresh corn began to be commercialized in the early 20th century, which promoted its cultivation worldwide [8]. Each hectare of corn can harvest about 600 kg of corn bracts; thus, the annual yield of fresh corn bracts worldwide is huge [9]. In recent years, as people have become more interested in natural active substances, fresh corn bracts have gradually received attention. Some studies show that fresh corn bracts are rich in flavonoids, anthocyanins, polysaccharides, dietary fiber, potassium, silicon, magnesium, selenium and other trace elements [10], of which dietary fiber accounted for the largest proportion [11]. A preliminary study shows that the proportion of insoluble dietary fiber in fresh corn bracts (FCB-IDF) is as high as 92%, while the proportion of soluble dietary fiber is only about 6%, which could not meet the requirements of dietary balance. The suitable dietary fiber composition is that soluble fiber accounts for more than 10% of the total dietary fiber content, as proposed by American scholars [12].

Dynamic high-pressure micro-fluidization (DHPM) technology was adopted to modify dietary fiber to reduce its fiber size and improve its physical, chemical and functional properties, and has become a good physical modification measure [13]. The modified dietary fiber from fresh corn bracts (FCB-MDF) with good physical and chemical properties was obtained through DHPM treatment in our previous study [11]. This paper intends to further investigate the effect of modified dietary fiber on the obesity characteristics, intestinal microflora distribution and short-chain fatty acids (SCFAs) of C57BL/6 mice that are fed a high-fat diet. The results of the study will provide a theoretical basis for improving the utilization rate of fresh corn bracts and developing healthy food that is rich in dietary fiber.

## 2. Results

### 2.1. Changes in Food Intake, Water Intake, Body Weight and Fecal Excretion of Mice

**Figure A1:**
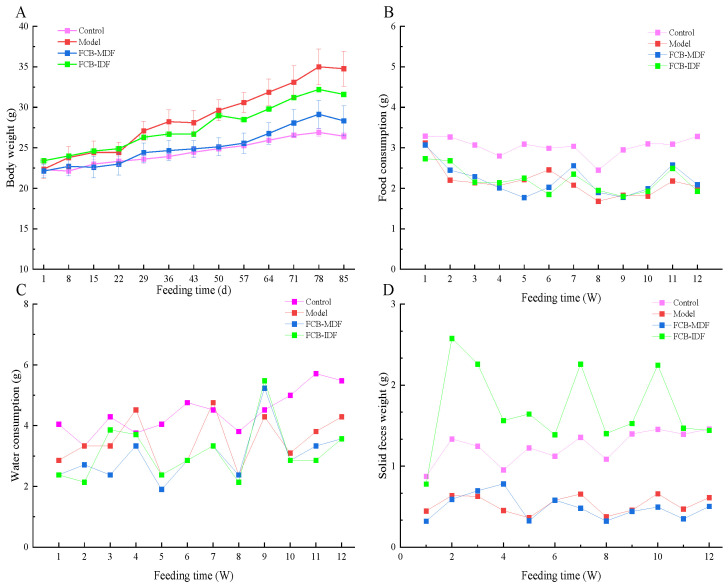
Effects of FCB-MDF on body weight, food intake, water consumption and solid feces in mice. Data are expressed as means ± standard deviations (n = 9).

As shown in Figure A1, compared with the high-fat feed groups, the mice in the control group consumed a larger amount of feed, and no significant difference (*p* > 0.05) was observed among the high-fat feed groups. The water consumption and fecal excretion were basically similar. The model group had the highest water consumption, and the FCB-IDF group had the largest fecal excretion in the high-fat feed groups. In addition, there was no significant difference (*p* > 0.05) in the initial body weights of the four groups of mice. With the intragastric intervention of FCB-IDF and FCB-MDF, the weight gain of the mice was inhibited. The weights of the mice in the FCB-MDF group had no significant difference from those in the control group since the fourth week (*p* > 0.05). Although the average weight of the mice in the FCB-IDF group decreased during the whole feeding period, there was no significant difference from that in the model group (*p* > 0.05). At the experimental end, the weights of the mice in the control group, model group, FCB-MDF group and FCB-IDF group were 26.43 ± 0.43 g, 34.76 ± 2.16 g, 28.33 ± 1.86 g and 31.69 ± 0.7 g, respectively. No significant difference was observed between the FCB-MDF group and the control group (*p* > 0.05).

### 2.2. Effect of FCB-MDF on Glucose Metabolism in Mice

**Figure 1:**
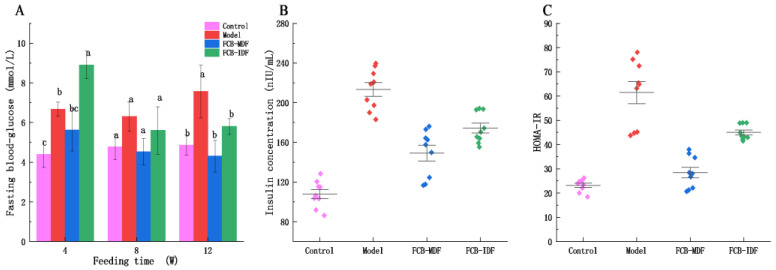
Effect of FCB-MDF on glucose metabolism in mice. (**A**) Fasting blood glucose level in mice at different feeding time periods; (**B**,**C**) scatter spacing diagram of insulin resistance in mice after 12 weeks of intervention. Data are expressed as means ± standard deviations (n = 9). Different superscript letters (a–c) denote statistically significant differences (*p* < 0.05).

It can be seen from Figure 1A that the fasting blood glucose level of the normal mice was stable between 4.40 and 4.87 mmol/L, which is significantly lower than that of the model group (*p* < 0.05). After 8 weeks of intervention, there was no significant difference in the blood glucose level between the FCB-MDF group and the control group (*p* > 0.05). After 12 weeks of intervention, no significant difference was observed between the FCB-IDF group, control group and FCB-MDF group (*p* > 0.05), and the blood glucose levels of these three groups were all significantly lower than that of the model group (*p* < 0.05). The results infer that both FCB-MDF and FCB-IDF had the effect of reducing fasting blood glucose in hyperlipidemic mice.

As shown in Figure 1B–C, the insulin concentration in the sera of the model group of mice was significantly higher than that of the other groups (*p* < 0.05), while the insulin concentration in the FCB-MDF group was lower than that of the FCB-IDF group (*p* > 0.05). By calculating the HOMA-IR index to measure the severity of insulin resistance in mice, it was found that the HOMA-IR indexes of the control group, model group, FCB-MDF group and FCB-IDF group were 27.47, 61.76, 27.50 and 44.00, respectively. The HOMA-IR index between the control group and the FCB-MDF group was similar, which was significantly lower than that of the model group (*p* < 0.05), indicating that FCB-MDF could improve insulin resistance in hyperlipidemic mice.

### 2.3. Effect of FCB-MDF on Lipid Metabolism in Mice

**Figure 2:**
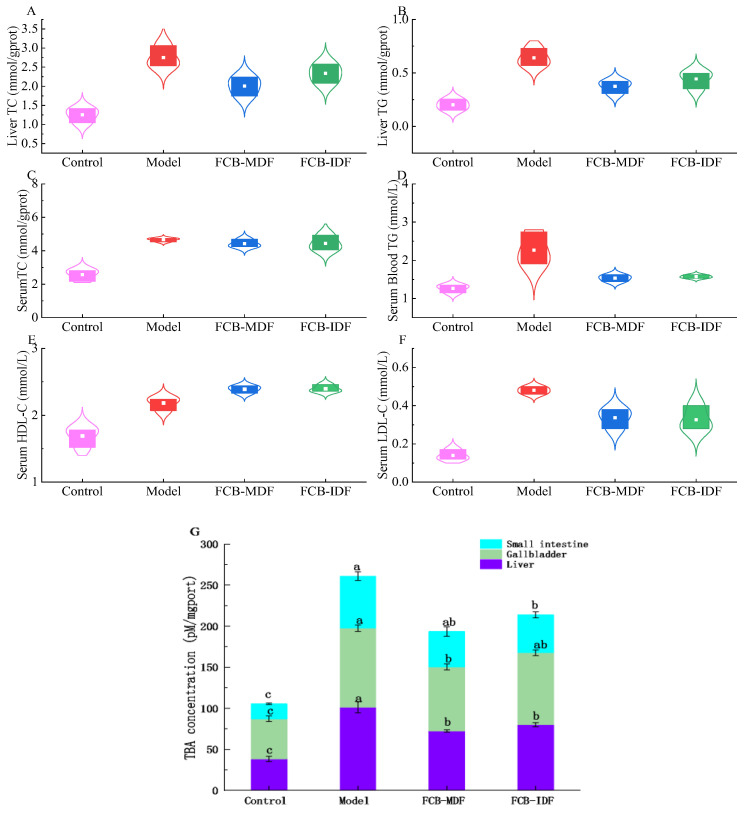
Effect of FCB-MDF on lipid metabolism in mice. (**A**,**B**) The concentrations of TC and TG in mouse liver; (**C**–**F**) the contents of TC, TG, HDL-C and LDL-C in mouse serum; (**G**) the TBA concentrations in mouse liver, gallbladder and small intestine. Data are expressed as means ± standard deviations (n = 9). Different superscript letters (a–c) denote statistically significant differences (*p* < 0.05). Abbreviations: TC, total cholesterol; TG, total triglyceride; HDL-C, high density lipoprotein; LDL-C, low density lipoprotein; TBA, total bile acid.

Abnormal blood lipids and liver triglyceride accumulation cause abnormal liver fat metabolism, thus affecting its function [14]. It can be seen from Figure 2A–F that a high-fat diet promoted the increase in the total triglyceride (TG), total cholesterol (TC), low density lipoprotein (LDL-C) and high density lipoprotein (HDL-C) in the sera, while after the intervention of FCB-MDF and FCB-IDF, the TG and LDL-C in the sera significantly decreased (*p* < 0.05), but there was no significant effect on the HDL-C and TC (*p* > 0.05). Both FCB-MDF and FCB-IDF reduced the accumulation of TG in the liver, and the effect of FCB-MDF was more obvious.

The concentration of total bile acid (TBA) represents the ability of the liver to metabolize cholesterol. It can be seen from Figure 2G that a high-fat diet caused a significant increase in the TBA in the livers and gallbladders of the mice (*p* < 0.05). Compared with the model group, the FCB-IDF and FCB-MDF feeding interventions significantly decreased the concentration of TBA (*p* < 0.05), indicating that the intervention might improve liver function and promote the liver metabolism of cholesterol, and the effect of FCB-MDF was better. In the small intestine, the concentration of TBA in the FCB-MDF and FCB-IDF groups also decreased significantly (*p* < 0.05), indicating that both FCB-MDF and FCB-IDF could inhibit TBA reabsorption, but no significant difference was observed (*p* > 0.05). However, the TBA concentration in the dietary fiber treatment group was still significantly higher compared with the control group (*p* > 0.05).

### 2.4. Effect of FCB-MDF on Oxidative Damage in Mice

**Figure 3:**
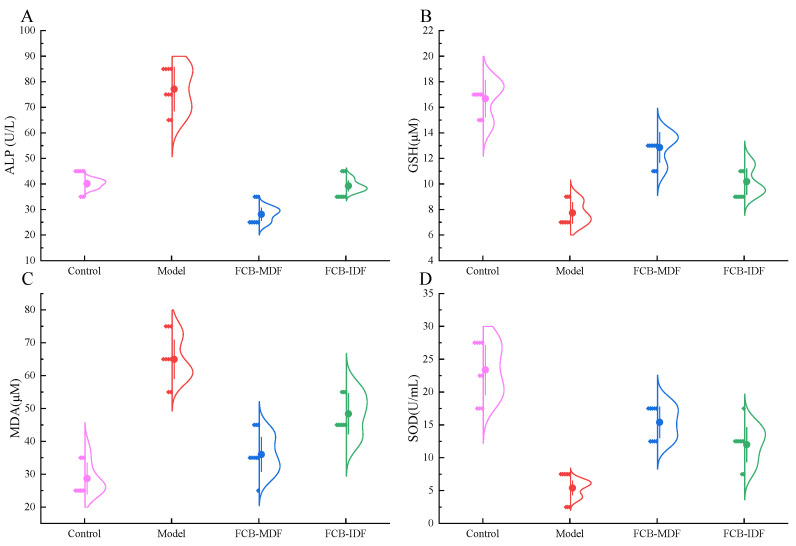
Effect of FCB-MDF on oxidative damage in mice. (**A**) ALP activity; (**B**) the GSH content; (**C**) the production of MDA; (**D**) SOD activity. Data are expressed as means ± standard deviations (n = 9). Abbreviations: ALP, alkaline phosphatase; GSH, glutathione; MDA, malondialdehyde; SOD, superoxide dismutase.

In order to further clarify the protective effect of FCB-MDF on liver tissue, the parameters related to oxidative stress and liver injury were measured. As shown in Figure 3, a high-fat diet significantly reduced the activity of antioxidant glutathione (GSH) and superoxide dismutase (SOD) (*p* < 0.05), increased the content of malondialdehyde (MDA) (*p* < 0.05), which was the end product of lipid oxidation, and also increased the alkaline phosphatase (ALP) activity (*p* < 0.05), which indicated impaired liver function. Compared with the control group, there was no significant difference in the SOD activity, ALP activity or MDA content in the FCB-MDF group (*p* > 0.05), while the GSH activity was significantly reduced (*p* < 0.05). FCB-IDF and FCB-MDF could not only inhibit the production of MDA, but also significantly increase the activity of SOD and the content of GSH, and reduce the activity of ALP, indicating that the liver damage was reduced.

### 2.5. Effect of FCB-MDF on Histopathology of Mice

**Figure 4:**
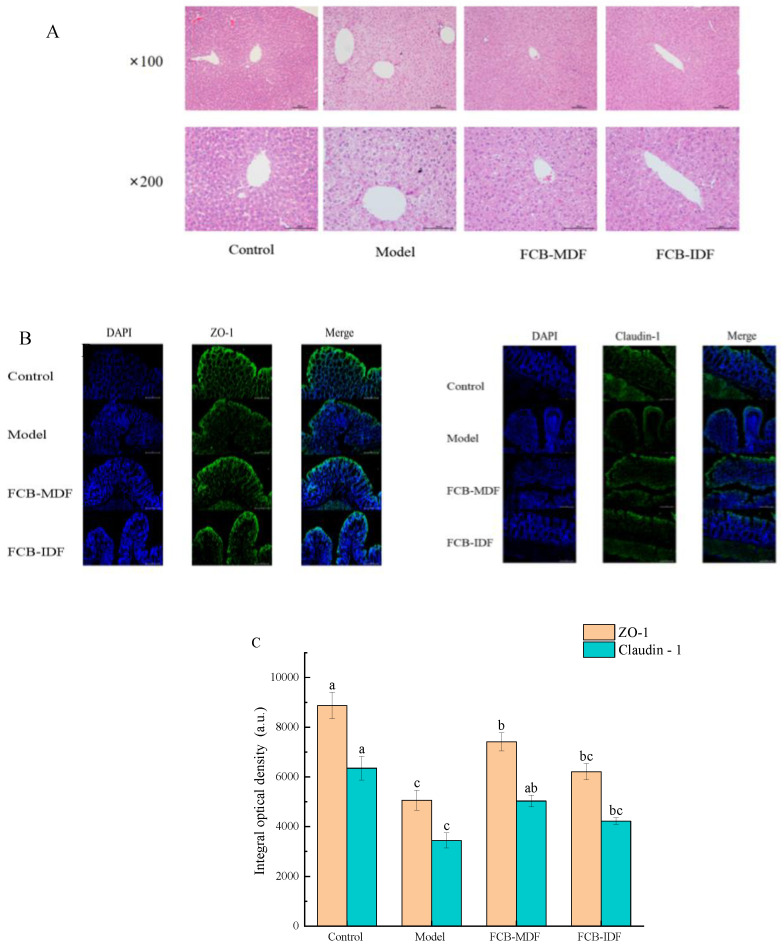
Effect of FCB-MDF on histopathology of mice. (**A**) The result of H&E staining; (**B**,**C**) the damage degree of tight junction proteins ZO-1 and Claudin-1 in mouse colon tissue. Data are expressed as means ± standard deviations (n = 9). Different superscript letters (a–c) denote statistically significant differences (*p* < 0.05). Abbreviations: ZO-1, Zona Occludens 1.

It can be seen from Figure 4A that after hematoxylin–eosin (H&E) staining, the hepatocytes of the mice in the control group took the central vein as the center, and were arranged neatly, with clear hepatocytes. The nucleus was located in the center of the cell, and was large and round, without fat vacuoles, indicating that the basic diet had no adverse effect on the livers of the mice. In the livers of the mice in the model group, the hepatocytes were arranged in disorder and the position of the nuclei was disordered. Meanwhile, the swollen nuclei of the hepatocytes were squeezed to the periphery, with a large amount of lipid deposition. The hepatocytes were filled with fat droplets of different sizes, forming a large number of fat vacuoles. After the intragastric administration of FCB-IDF and FCB-MDF, the number of vacuoles and ballooning of hepatocytes in the livers of the mice were significantly reduced (*p* < 0.05), and the amount of fat drops in the livers was significantly reduced (*p* < 0.05), indicating that both of them improved the liver tissue structures of the high-fat-diet mice and reduced the fat deposition caused by the high-fat diet.

The damage degree of tight junction proteins ZO-1 (Zona Occludens 1) and Claudin-1 in mouse colon tissue was investigated using fluorescence detection. As seen in Figure 4B,C, compared with the control group, the expression levels of ZO-1 and Claudin-1 in the colon tissues of the model group of mice were significantly reduced (*p* < 0.05). The expression of tight junction protein in the colon tissues of mice in the FCB-MDF group was significantly higher than that in the model group (*p* < 0.05), and there was no significant difference in the expression of Claudin-1 between the FCB-MDF group and the control group (*p* > 0.05). Compared with the model group, the expression levels of ZO-1 and Claudin-1 in the colon tissues of mice in the FCB-IDF group were not significantly increased (*p* > 0.05). This indicates that FCB-MDF could effectively repair the intestinal barrier damage caused by a high-fat diet.

### 2.6. Effect of FCB-MDF on Short-Chain Fatty Acids in Mice

**Figure 5:**
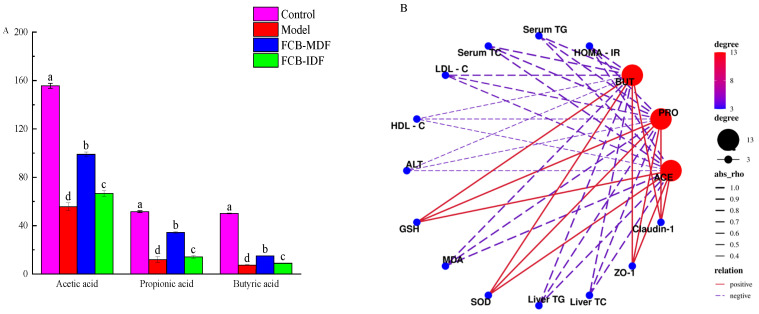
SCFAs in the cecum contents of mice after 12 weeks of intervention (**A**) and their network correlation diagram with various biochemical indicators (**B**). Data are expressed as means ± standard deviations (n = 9). Different superscript letters (a–d) denote statistically significant differences (*p* < 0.05). Abbreviations: TG, total triglyceride; TC, total cholesterol; LDL-C, low density lipoprotein; HDL-C, high density lipoprotein; ALP, alkaline phosphatase; TBA, total bile acid; GSH, glutathione; MDA, malondialdehyde; SOD, superoxide dismutase; ZO-1, Zona Occludens 1; BUT, butyric acid; PRO, propionic acid; ACE, acetic acid.

As shown in Figure 5, a long-term high-fat diet led to the most obvious decrease in the butyric acid level in the cecum contents of the mice. The butyric acid content of the model group was only 14.7% of that of the control group, of which acetic acid was the least affected, and was only 35.8% of that of the control group. Compared with the model group, FCB-MDF significantly increased the concentration of acetic acid, propionic acid and butyric acid (*p* < 0.05). FCB-IDF restored the concentrations of acetic acid, propionic acid and butyric acid to 42.8%, 27.3% and 17.8% of the control group, respectively, while FCB-MDF returned to 63.6%, 66.7% and 29.9% of the control group, respectively. However, the SCFAs of the dietary fiber group were still significantly lower than those of the control group. The Spearman correlation analysis showed that there was a strong positive correlation between the antioxidant active substances and the concentration of SCFAs, while the HOMA-IR, the concentration of TC and TG in the serum and liver and the concentration of MDA were strongly negatively correlated with the concentration of SCFAs, the concentration of LDL-C was negatively correlated with the concentration of SCFAs and the concentration of HDL-C and ALP was weakly negatively correlated with the concentration of SCFAs. These findings suggest that SCFAs play a key role in the prevention of metabolic syndrome by regulating lipid balance and inhibiting oxidative stress.

### 2.7. Effect of FCB-MDF on Intestinal Microflora in Mice

#### 2.7.1. Basic Distribution of Species in Each Treatment Group

**Figure A2:**
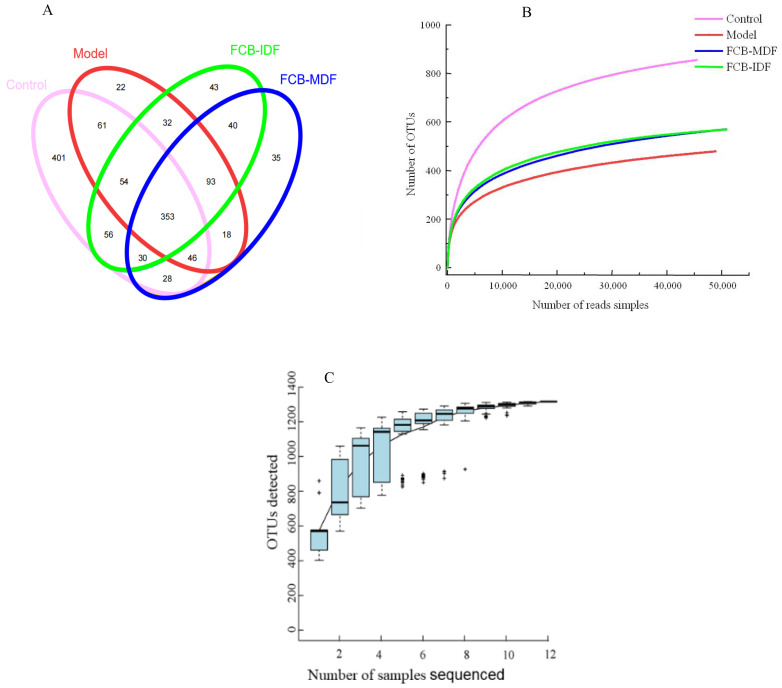
Venn diagram based on OTUs (**A**), sparse curve (**B**) and species accumulation curve (**C**) of different fecal samples.

A total of 51037 effective sequences were obtained from 12 stool samples (the cecal contents of three mice in the same treatment group were randomly merged into one sample). The average effective sequences in the control group, model group, FCB-MDF and FCB-IDF groups were 46,388 ± 2581, 46,764 ± 2778, 44,255 ± 2577 and 32,716 ± 886, respectively. When the similarity was greater than 97%, the numbers of operational taxonomic units (OTUs) of the four groups were 810.00 ± 38.97, 500.33 ± 40.10, 516.00 ± 97.87 and 490.00 ± 76.53, respectively. Figure A2A shows a Venn diagram of the OTUs that are shared by multiple groups or only unique to one group. There were 353 OTUs in all the groups, and 401, 22, 43 and 35 OTUs in the control group, model group, FCB-IDF and FCB-MDF groups, respectively.

#### 2.7.2. Comparative Analysis of Mouse Fecal Microflora Based on Alpha Diversity

**Figure A3:**
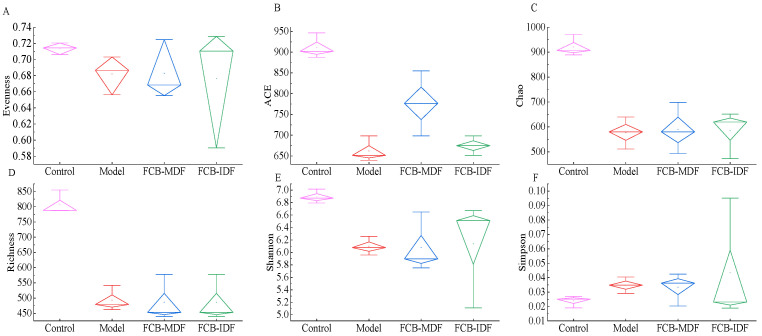
Mouse fecal microflora comparison of α diversity analysis.

As seen from the dilution curve and the species accumulation curve shown in Figure A2B,C, with the increase in the number of sequenced samples, the curve mean line rose rapidly and then tended to be flat. The continued increase in the number of sequenced samples only generated a small number of new OTUs, which indicates that the number of sequenced samples covers the vast majority of species information in the samples and could meet the experimental requirements. The depth index of sample sequencing was 0.9969, indicating that the sequencing results reflect the real situation of species based on the fecal samples. The effect of different treatment groups on the alpha diversity index of mouse microflora is shown in Figure A3. The evenness represented the uniformity of microflora, the Chao and richness indexes represented the abundance of microflora and the Shannon, Simpson and ACE indexes were used to estimate the species richness of mouse fecal flora. Compared with the control group, the uniformity, abundance and richness of the model group, FCB-IDF group and FCB-MDF group performed overall decreasing trends, which shows that a high-fat diet led to the decrease in the alpha diversity of intestinal microflora in the mice.

#### 2.7.3. Comparative Analysis of Mouse Fecal Microflora Based on Beta Diversity

**Figure A4:**
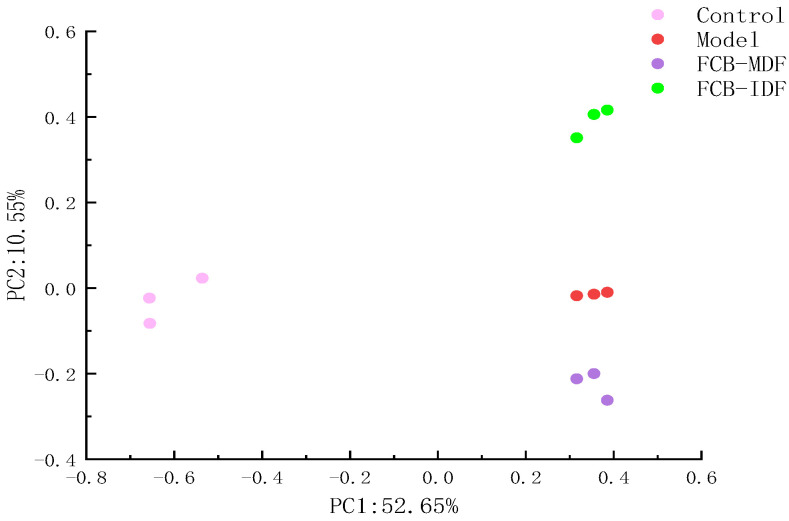
PCoA analysis of mouse fecal microflora composition.

The principal coordinate analysis (PCoA) was performed based on the unweighted UniFrac distance. There were obvious distances between the four groups in Figure A4, which were gathered at different positions of the coordinates, indicating that different treatments led to differences in the microflora structure. The distance between the FCB-IDF group and the model group was relatively close, indicating that the microflora structure was relatively similar, while the distance between the FCB-MDF group and the model group was relatively far. The contribution rates of PC1 (*X*-axis) and PC2 (*Y*-axis) were 52.56% and 10.55%, respectively. There was a certain difference between the control group and the model group, FCB-IDF group and FCB-MDF group, indicating that a high-fat diet led to changes in the microflora structure. PC1 and PC2 distinguished the model group, FCB-IDF group and FCB-MDF group to a certain extent, indicating that the intake of dietary fiber led to changes in the intestinal microflora, and the distance between the FCB-MDF group and model group was farther, which implies a greater gap.

#### 2.7.4. Distribution Results of Each Treatment Group of Species at the Level of Phyla and Genus

**Figure 6:**
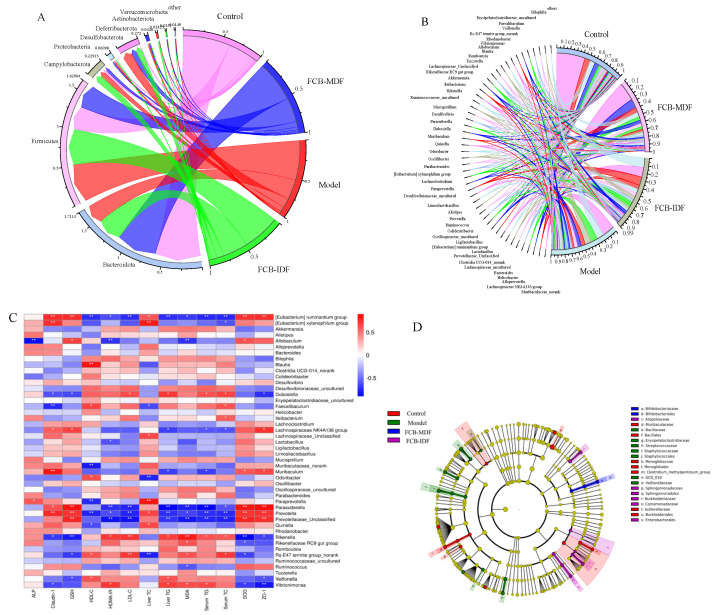
Effect of FCB-MDF on intestinal microflora in mice. (**A**) Chord chart of phylum level; (**B**) chord chart of genus level; (**C**) heatmap of the relationship between the proportion of microbiota at the genus level and various biochemical indicators related to obesity. The colors range from blue (negative correlation) to red (positive correlation). Significant correlations are noted by * *p* < 0.05 and ** *p* < 0.01. (**D**) LEfSe analysis of fecal microflora structure.

The distribution of mouse fecal microflora at the phyla level is shown in Figure 6A. The mouse fecal microflora at the phyla level mainly consisted of *Bacteroidota*, *Firmicutes*, *Campylobacterota*, *Proteobacteria*, *Desulfobacterota*, *Actinobaciota*, *Deferribacterota*, *Verrucomicrobiota* and other components. The main dominant bacteria were *Bacteroidota* and *Firmicutes*, accounting for more than 60% of the fecal bacteria. Compared with the control group, *Firmicutes*, *Campylobacterota*, *Desulfobacterota* and *Actinobaciota* in the model group all increased significantly. The most obvious increase was *Desulfobacterota*, which was 1092% of the control group. The second was *Actinobaciota*, which was 258.4% of the control group. The most obvious decreases were *Deferribacterota* and *Bacteroidota*, which were 26.4% and 42.7% of the control group, respectively. After the intragastric administration of FCB-MDF, *Bacteroidota*, *Firmicutes* and *Campylobacterota* recovered to 88.1%, 97.8% and 93.2%, respectively, of the control group, while *Desulfobacterota* decreased to 59.7% of the model group. After FCB-IDF intragastric intervention, *Bacteroidota*, *Firmicutes* and *Campylobacterota* were 53.6%, 149.5% and 240.7%, respectively, of the control group, while *Desulfobacterota* decreased to 57.8% of the model group.

At the genus level, the fecal microflora of each mouse mainly included *Muribaceae*, *Lachnospiraceae*, *Alloprevotella*, *Helicobacter*, *Bacteroides*, *Clostridia*, *Prevotellaceae-Enclassified*, *Lactobacillus*, the *[Eubacterium] ruminantium* group, *Oscillospiraceae* and other components (Figure 6B). Among the bacterial populations that accounted for more than 1%, *Prevotellaceae_Unclassified* decreased the most, and was only 0.12% of the control group, and *Helicobacter* increased the most, and was 267.4% of the control group. *Muribaculaceae_norank*, the *Lachnospiraceae NK4A136* group, *Alloprevotella*, *Ligilactobacillus*, *Prevotellaceae_Unclassified*, *Lactobacillus*, the *[Eubacterium] ruminantium* group, *Ruminocus* and *Prevotella* all decreased to different degrees. *Helicobacter*, *Lachnospiraceae_uncultured*, *Oscillospiraceae_Uncultured* and *Colidextribacter* increased. FCB-MDF supplements reduced the proportion of *Helicobacter*, *Lactobacillus* and *Colidextribacter*, and increased the proportion of *Muribaculaceae_norank*, *Alloprevotella*, *Clostridia UCG-014_norank*, *Oscillospiraceae_Uncultured* and *Ruminocus*. However, after the intragastric administration of FCB-IDF, the proportions of the *Lachnospiracea NK4A136* group, *Lactobacillus*, *Oscillospiracea_uncultured*, *Colidextribacter*, and *Ruminocus* increased, while the proportions of *Alloprevotella*, *Bacteroides* and *Helicobacter* decreased.

The Spearman correlation analysis (Figure 6C) of the microflora and biochemical indicators at the genus level showed that the microflora was closely related to the biochemical indicators; accounting for more than 1% of the total was *Murbaculaceae_norank*, *Alloprevotella*, *Lachnospiraceae_Uncultured*, the *[Eubacterium] ruminantium* group, *Colidextribacter*, *Ruminococcus* and *Prevotella*, where the *[Eubacterium] ruminantium* group, *Alloprevotella*, *Muriaculaceae_Norank*, *Ruminococcus* and *Prevotella* played active roles in resisting the damage caused by the high-fat diet, while *Colidextribacter* and *Lachnospiracea_Uncultured* might promote the development of metabolic syndrome. Further analysis of the specific bacteria in the different groups revealed that (Figure 6D), at the class level, the specific bacteria in the control group were *Bacillales*, *Monoqlobacaeae* and *Burkholderiales*, the specific bacteria in the model group were *Staphylococcaceae*, the specific bacteria in the FCB-MDF group were *Bifidobacteriaceae* and the specific bacteria in the FCB-IDF group were *Sphingomonadaceae* and *Enterobacters*. At the order level, the unique microflora of the control group included *Murbaculaceae*, *Monolobales*, the *Clostridium metapentosum* group and *Sutterelaceae*; the unique microflora of the model group included *Bacillaceae*, *Erysipelatoclostridiaceae*, *Streptococcus*, *Staphylococcae*, *UCG010* and *Veillonellaceae*; the unique microflora of the FCB-MDF group was *Bifidobacteriales* and the unique microflora of the FCB-IDF group included *Atopobiaceae*, *Sphingomonadales*, *Burkholderiaceae* and *Commonadaceae*.

## 3. Discussion

There are various complex metabolic pathways in the human body, among which the insulin–glucagon system is directly related to the human glucose level. Research shows that a high-fat diet could induce energy metabolism dysfunction and cause insulin resistance [2,15]. Insulin resistance is not only a key component of metabolic syndrome, but also the most important pathophysiological feature of many diseases in pre-diabetes. Normal levels of insulin cannot stimulate the response of target cells [16], which, in turn, promote the pancreas to produce more insulin, causing the overload of the pancreas to accelerate the decline in pancreatic cell function. When the liver, an important organ involved in glucose and lipid metabolism, creates insulin resistance, this leads to blocked gluconeogenesis and impaired cellular function [17]. With the increase in fat intake, the free fatty acids produced by hydrolysis also increase, and triglycerides gradually accumulate in the liver. The overload of triglycerides in hepatocytes could induce a large number of inflammatory factors and produce oxidative stress [18,19,20]. In this study, mice on a high-fat diet showed weight gain, increased cholesterol and triglyceride content in the body and increased fasting blood glucose level and insulin content, resulting in oxidative, fatty liver and intestinal damage. Compared with the model group, the obesity and specific injury symptoms of the mice were significantly relieved after the intervention of FCB-MDF, and the effect was better than that of FCB-IDF.

The interaction between dietary components and intestinal microorganisms shaped the intestinal microflora and affected the host metabolism [21]. Intestinal microflora plays an important role in the development of human health as one of the largest organs [22]. Under normal conditions, intestinal microflora participates in the fermentation of some macromolecular polysaccharides that cannot be decomposed by endogenous enzymes, and has the functions of promoting the production of SCFAs, synthesizing vitamins, maintaining the integrity of intestinal mucosa and inhibiting the proliferation of harmful bacteria in the intestine [23]. The main function of dietary fiber is to serve as the fermentation substrate of intestinal microorganisms, regulate the proportion of intestinal microorganisms and promote the production of SCFAs. Therefore, it was speculated that FCB-MDF might reduce obesity and related symptoms by regulating intestinal microflora. The experimental results show that a high-fat diet significantly reduced the abundance and richness of intestinal microflora in the mice. FCB-MDF and FCB-IDF positively changed the proportion of intestinal microflora.

It was considered that abnormal lipid metabolism was related to changes in the proportion of *Firmicutes* and *Bacteroidota* in the intestines of animals [24]. Li et al. [25] reported that a high-fat and high-fructose diet led to the increase in *Firmicutes* and the decrease in *Bacteroidota*, while the experimental results obtained by Zheng et al. showed that the abundance of *Firmicutes* in obese mice was lower and the content of *Bacteroidota* was higher [26]. The results of our study show that a high-fat diet increased the proportion of *Firmicutes/Bacteroidota* (F/B) and led to the abundance of *Desulfobacterota*. Due to the fact that *Firmicutes* could produce more recoverable energy than *Bacteroidota*, the relatively high abundance of *Firmicutes* led to increased calorie absorption and promoted obesity. FCB-IDF and FCB-MDF helped to maintain the F/B ratio at a low level, which reduced their blood lipid levels. Some studies believed that the supplementation of specific *Desulfovibrio vulgaris* could promote the production of acetic acid and improve non-alcoholic fatty liver disease [27]. However, the excessive proliferation in *Desulfobacterota* would promote the expression of receptor CD36, which is involved in lipid absorption, thereby regulating the lipid absorption capacity of the host and affecting obesity [28]. In this study, the proportion of *Desulfobacterota* in the intestines of mice fed with a high-fat diet increased significantly, but the intervention of FCB-MDF significantly reduced its proportion.

Additionally, FCB-MDF promoted the proliferation of *Murbaculaceae_norank*, *Alloprevotella*, *Clostridia UCG-014_norank*, *Oscillospiraceae_uncultured* and *Ruminococcus*, and reduced the proportion of *Helicobacter*, *Lactobacillus* and *Colidextribacter*. Some studies have shown that *Muriaculacea* was considered to be a group of bacteria that degraded carbohydrates and produced butyrate. Its proportion in the intestinal microflora was significantly negatively correlated with the level of blood lipids [29], and it was also involved in the formation of the mucosal layer and the improvement in the barrier function in the colon. The decrease in *Muribagulaceae* might cause the increase in blood lipids, which is not conducive to colon health [30]. *Alloprevotella* is another genus in the *Prevotellaceae* family. It is a Gram-negative, specific anaerobic bacteria with the ability to ferment carbohydrates and produce SCFAs (acetate and butyrate). Many studies have shown that its abundance was negatively correlated with obesity, diabetes, cardiovascular disease and metabolic syndrome [31]. The proliferation of *Clostridia UCG-014_Norank* and *Oscillospiracea_uncultured* could improve the immune function of mice [32], and the proliferation of *Ruminococcus* could improve the intestinal barrier function [33]. *Helicobacter* is a bacterial genus that mainly lives in the upper digestive tract and is generally considered to be infectious and pathogenic. It is considered as a Class I carcinogen, which invades and colonizes the host, controls the immune system and drives the key marker genes of cancer, such as promoting the inflammation–cancer reaction, destroying the anti-tumor immunity and up-regulating the cell proliferation signal, leading to the occurrence of gastric MALT lymphoma and gastric adenocarcinoma [34]. *Colidextribacter* is closely related to the parameters related to hyperlipidemia. The proportion of *Lactobacillus* also decreased, which might be due to the fact that FCB-MDF is not suitable as the substrate. It is noteworthy that in the FCB-MDF group, *Bifidobacteriaceae* was the intergroup difference bacteria, which indicates that the environment where FCB-MDF exists is extremely suitable for *Bifidobacteriaceae* proliferation. As is known, *Bifidobacteriaceae* could attach to the intestinal mucosa and form a biological barrier as probiotics. It protects the intestinal mucosa and could seize the proliferation sites of pathogenic bacteria. Meanwhile, *Bifidobacteriales* could produce acid substances such as lactic acid and acetic acid, inhibit the growth of harmful microorganisms and promote intestinal peristalsis [35]. It could also synthesize a variety of vitamins, decompose the free radicals produced, inhibit the production of peroxides and slow down aging.

The SCFAs are produced after the fermentation of dietary fiber by intestinal microorganisms. SCFAs improve glucose homeostasis by regulating the function of skeletal muscle, liver and adipose tissue and the sensitivity of target cells to insulin [36], promote the proliferation of intestinal epithelial cells and the expression of tight protein [37] and act on the Ffar2 receptor of the surface of intestinal Treg cells to inhibit histone deacetylase, thereby promoting the proliferation of Treg and reducing inflammation [38]. For example, in the liver, propionate could inhibit 3-hydroxy-3-methylglutaryl coenzyme, a reductase used to regulate host lipid metabolism, inhibit oxidative stress and pro-inflammatory cytokine production [38]. Butyrate provides nicotinamide adenine dinucleotide phosphate for GSH synthesis and induces apoptosis with the increased expression of oxidative pathogens [39]. Therefore, the SCFAs produced by the fermentation of dietary fiber are very important for health. In this study, FCB-MDF significantly increased the concentration of SCFAs, and there was a strong positive correlation between the concentration of SCFAs and antioxidant active substances, while the HOMA-IR, the concentration of TC and TG in serum and liver and the concentration of MDA were strongly negatively correlated with the concentration of SCFAs; thus, the increase in the concentration of SCFAs was closely related to the reduction in obesity in mice.

Tight junction proteins (including ZO-1, Occludin and Claudin-1) are the main determinant of intestinal barrier function to maintain mucosal permeability, and the role they play in intestinal epithelial cells is the most important [40]. The results of our study show that a high-fat diet causes damage to the intestinal barrier, which is mainly manifested by the decreased expression of tight junction protein; this would induce microbial pathogens and harmful bacterial substances (such as LPS) to enter the blood [41], and aggravate chronic low-grade inflammation. However, FCB-MDF increases the expressions of ZO-1 and Claudin-1, repairs the intestinal barrier and possibly slows down the inflammatory effect.

In summary, the DHPM-modified FCB-IDF is a natural dietary fiber with probiotic effects, which can be used as a substrate for intestinal microflora fermentation to change the composition of intestinal microorganisms and produce SCFAs to alleviate oxidative stress, fatty liver and intestinal damage caused by a high-fat diet in mice.

## 4. Materials and Methods

### 4.1. Materials

Fresh corn bracts were provided by Jiangsu Jia’an Food Technology Co., Ltd. (Nantong, China); high-fat feed (60 kcal% fat) was purchased from Jiangsu Synergy Bioengineering Co., Ltd. (Nanjing, China); pentobarbital sodium was purchased from Shanghai Fude Chemical Co., Ltd. (Shanghai, China); the Mouse Insulin ELISA kit was from Shanghai Haoyang Biotechnology Co., Ltd. (Shanghai, China); the total glutathione assay kit, total superoxide dismutase activity assay kit, malondialdehyde ELISA kit, BCA protein assay kit and cell lysate were all purchased from Shanghai Biyuntian Biotechnology Co., Ltd. (Shanghai, China) and the OCT embedment agent was purchased from Beijing Solebo Technology Co., Ltd. (Beijing, China). Thirty-six 7-week-old male C57BL/6J mice without specific pathogen (SPF) at room temperature were purchased from Henan Scribes Biotechnology Co., Ltd. (Anyang, China).

### 4.2. Extraction and Modification of Insoluble Dietary Fiber from Fresh Corn Bracts

The fresh corn bracts were dried and crushed, and the insoluble dietary fiber (FCB-IDF) was extracted according to AOAC 985.29 enzymatic-gravimetric method in foods [42]. The obtained product was modified via dynamic high-pressure micro-fluidization; the solid–liquid ratio was 1.5 g/100 mL and the treatment pressure was 12,900 PSI. The final modified fiber was named FCB-MDF, with the average particle size of 3.06 ± 0.045 μm.

### 4.3. Animal Experiment Design

Thirty-six healthy 7-week-old male C57BL/6J mice were raised under specific conditions (12/12 h light and dark cycle, humidity 60 ± 5%, room temperature within 20–24 °C), with nine mice in each cage. In the adaptation stage, all mice could freely eat (standard rodent feed) and drink water. All experimental protocols were carried out in strict accordance with the guidelines of the National Institutes of Health for the care and use of experimental animals (NIH publication No. 8023, revised in 1978). After 7 days of adaptive feeding, the mice were randomly divided into the following 4 groups, with 9 mice in each group. These included control group (standard rodent feed + 0.9% normal saline 200 mg/kg•bw), model group (60 kcal% high fat feed + 0.9% normal saline 200 mg/kg•bw), FCB-IDF treatment group (60 kcal% high fat feed + FCB-IDF 200 mg/kg•bw) and FCB-MDF treatment group (FCB-MDF, 60 kcal% high fat feed + FCB-MDF 200 mg/kg•bw). All treatments were tested continuously for 85 days. The body weight, food intake, water consumption and defecation of the mice were recorded every week. The fasted blood glucose was measured every 4 weeks. After the last treatment, the mice fasted overnight. All mice were anesthetized in a sealed device filled with isoflurane gas, and then euthanized via cervical dislocation to obtain samples of blood, liver, gallbladder, colon, small intestine, cecum contents, feces and fat.

### 4.4. Determination of Biochemical Indicators in Sera and Plasma of Mice

After slowly dissolving the serum samples from each treatment group and storing them in a refrigerator at −80 °C and at 4 °C, the sera were sequentially placed into a Hitachi 7100 automatic biochemical analyzer for detection. The contents of TG, TC, LDL-C, and HDL-C and the activity of ALP in the sera were, respectively, obtained.

The insulin doses were determined according to the instructions of the mouse insulin ELISA kit. The insulin resistance level was calculated according to the following Formula (1).

(1) $$HOMA-IR\;=\;\frac{C_{1}\times C_{2}}{22.5}\;$$

where C_1_ is the blood glucose concentration, mmol/L; C_2_ is the blood insulin concentration, mU/L.

The collected blood was centrifuged at 600 g for 10 min to obtain the supernatant. After diluting it to a certain proportion, the activity of SOD and the contents of MDA and GSH in plasma were measured according to the instructions of the kit.

Part of the weighed liver tissue was homogenized by adding 0.2–0.3 mL of PBS in an ice water bath, and the homogenate was taken to detect TC and TG in the liver tissue according to the instructions of the kits.

### 4.5. Determination of Bile Acid Content in Livers, Gallbladders and Small Intestines of Mice

The contents of TBA in livers, gallbladders and small intestines were, respectively, determined. The liver tissue (100–150 mg) was homogenized with 0.5 mL PBS, then added with 1 mL of ethanol to shake and extract for 1 h, centrifuged at 3500 rpm for 5 min to obtain the supernatant, and then extracted with 1.5 mL of ethanol solution for 1 h to obtain the supernatant and determine the TBA content according to the official instructions of the kit. Similar to the extraction of TBA from liver tissue, 3 mL PBS was added to the whole small intestine tissue for tissue homogenization, and then 3 mL ethanol solution was used to extract the supernatant twice to determine the content of TBA. The gallbladder tissue was diluted with 5 mL PBS and the TBA was determined directly.

### 4.6. Histopathological Analysis

#### 4.6.1. Histopathological Determination of Mouse Liver

The liver tissue fixed with 10% formalin was embedded in paraffin and cut into 4 μm thick slices. Two sections from each tissue were taken for H&E staining observation.

#### 4.6.2. Immunofluorescence Assay of Mouse Colon Tissue

The paraffin section of colon tissue was soaked in EDTA buffer (pH = 9) to repair the antigen, and then washed with PBS (pH = 7.4) 3 times, each time for 5 min. A circle of filter paper was drawn around the section with an immunofluorescence pen to prevent the loss of antibody, 3% H_2_O_2_ and PBS (pH = 7.4) were added on the section, then 100μL of cell staining buffer was dripped after 8 min and incubated at room temperature for 2 h. One section was dripped with ZO-1 monoclonal antibody solution (1 μg/mL, 0.3% trironX-100, prepared with PBS with pH = 7.4), another slice was dripped with Claudin-1 monoclonal antibody solution (1 μg/mL, 0.3% trironX-100, prepared with PBS with pH = 7.4), and after the tissue sections were covered, they were incubated overnight at 4 °C. They were taken out the next day, placed at room temperature for 1 h, washed with PBS (pH = 7.4) 3 times, each time for 5 min, then the liquid around the section was dried with filter paper, and they were dripped with fluorescein isothiocyanate-labeled sheep anti-rabbit fluorescent secondary antibody, and incubated at room temperature in dark for 2 h. The above washing steps were repeated, and then 4’, 6-diamino-2-phenylindole diacetate (DAPI) nuclear dye solution was added, respectively, and incubated in dark at room temperature for 10 min; 5 μL anti-fluorescence quenching agent was dripped for seal, then observed under the microscope for collecting images, respectively. Image J software was used to analyze the optical density, and the blank group was used as the control to calculate the relative fluorescence intensity of other groups.

### 4.7. Determination of Short-Chain Fatty Acids in Mouse Feces

According to the previous method [43], 400 μL of sample or standard solution was mixed with 400 μL 0.3 mg/mL of 2-ethylbutyric acid solution prepared by 0.2 M HCl, centrifuged at 15,000 rpm for 5 min, and the supernatant was obtained for analysis. The amount of sample added was 1 μL. The chromatographic column was HP-INNOWAX column (30 m × 0.25 mm × 0.25 μm, Agilent), the carrier gas was N_2_, the flow rate was 19.0 mL/min, and the air, H_2_ and N_2_ makeup flow rates were 260, 30 and 30 mL/min, respectively. The initial column temperature was 100 °C. After holding for 1 min, the column temperature increased to 180 °C at the rate of 5 °C/min, and remained at 180 °C for 4 min.

### 4.8. Determination of Intestinal Microflora in Mice

According to the manufacturer’s instructions, the rapid DNA extraction kit was used to extract the intestinal microbiota DNA from the feces of mice, then the quantity and quality of extracted DNA were checked via 1% agarose gel electrophoresis. Specific primers with barcode were synthesized according to the specified sequencing region. All samples were carried out according to the formal experimental conditions, with three replicates for each sample. The PCR products of the same sample were mixed and detected via 2% agarose gel electrophoresis. The PCR products were cut and recovered by using the Axy Prep DNA gel recovery kit and eluted via Tris HCl; 2% agarose electrophoresis detection. With reference to the preliminary quantitative results of electrophoresis, the PCR products were used with Quanti Fluor ™—The ST blue fluorescence quantitative system for detection and quantification, and then the corresponding proportion was mixed according to the sequencing quantity requirements of each sample. The library of Illumina PE250 was constructed and sequenced. The PE reads obtained using Illumina PE250 sequencing were spliced according to the overlay relationship, and the sequence quality was controlled and filtered at the same time. After the samples were differentiated, OTU cluster analysis and species taxonomy analysis were performed. Based on the OTU cluster analysis results, the OTU diversity index analysis and the sequencing depth detection were performed on the OTU. Based on taxonomic information, the statistical analysis of community structure was carried out at each classification level. Principal coordinates analysis (PCoA) was applied to examine dissimilarities in community composition, and microbiota abundances were constructed based on the unweighted UniFrac distance metric. Heatmap analysis was conducted to investigate correlations between gut microbiota and biochemical indexes related to obesity. 

### 4.9. Statistics Analysis

All data are expressed as mean ± standard deviation. SPSS19.0 software was used to analyze the statistical significance between different treatment groups through Duncan’s multiple range test, and the significance threshold was set at *p* < 0.05. The correlation between obesity phenotype and gut microbiota composition was calculated using Spearman correlation analysis; when *p* < 0.05, the correlation was considered significant.

## 5. Study Limitations

This study preliminarily reveals the effects of FCB-MDF on lipid metabolism and gut microbiota in high-fat-diet mice. The internal correlation analysis is not yet comprehensive, and further in-depth research and exploration are needed in the future.

## 6. Conclusions

In this study, the dynamic high-pressure micro-fluidization (DHPM) technology was adopted to modify the insoluble dietary fiber from fresh corn bracts. Then, the effects of FCB-MDF on the pathology of obesity, the distribution of microbiota and the production of SCFAs in mice that were fed a high-fat diet were studied. Under the given experimental method, the results show that FCB-MDF could slow down the obesity, glucose and lipid metabolism disorder and oxidative damage of mice caused by a high-fat diet, and promote the production of acetic acid, propionic acid and butyric acid, and the effect was significantly better than that observed when using unmodified dietary fiber. Additionally, FCB-MDF improved the proportion of different bacteria in the intestinal microflora of mice to a certain extent, and especially reduced the proportion of *Firmicutes* and *Bacteroidota*. Compared with the unmodified dietary fiber, FCB-MDF greatly promoted the proliferation of the beneficial bacteria *Bifidobacteriales* and had significant prebiotic functions. The fresh corn bract dietary fiber modified by DHPM seems to have the obvious potential to selectively change the distribution of intestinal microflora in mice, and it might be used as a potential food additive to treat intestinal microflora ecological imbalance or prevent obesity risk.

## Data Availability

The authors can provide the data if needed.

## Sample Availability

Samples of the compounds are available from the authors.
